# Using Social Media as a Research Tool for a Bespoke Web-Based Platform for Stakeholders of Children With Congenital Anomalies: Development Study

**DOI:** 10.2196/18483

**Published:** 2021-11-15

**Authors:** Marlene Sinclair, Julie E M McCullough, David Elliott, Paula Braz, Clara Cavero-Carbonell, Lesley Dornan, Anna Jamry-Dziurla, Ana João Santos, Anna Latos-Bieleńska, Ausenda Machado, Lucía Páramo-Rodríguez

**Affiliations:** 1 Institute of Nursing and Health Research Ulster University Northern Ireland United Kingdom; 2 Redburn Solutions Ltd Belfast United Kingdom; 3 Epidemiology Department National Institute of Health Doctor Ricardo Jorge Lisbon Portugal; 4 Rare Diseases Research Unit Foundation for the Promotion of Health and Biomedical Research in the Valencian Region Valencia Spain; 5 Department of Medical Genetics Poznan University of Medical Sciences Poznan Poland; 6 Public Health Research Centre National School of Public Health Nova University Lisbon Lisbon Portugal

**Keywords:** Facebook, YouTube, Twitter, social media, metrics, e-forum, congenital anomalies, coproduction, COVID-19

## Abstract

**Background:**

Limited research evidence exists on the development of web-based platforms for reciprocal communication, coproduction research, and dissemination of information among parents, professionals, and researchers. This paper provides learning and the outcomes of setting up a bespoke web-based platform using social media.

**Objective:**

This study aims to explore the establishment of a web-based, multicontextual research communication platform for parents and stakeholders of children with congenital anomalies using social media and to identify associated research and ethical and technical challenges.

**Methods:**

The ConnectEpeople e-forum was developed using social media platforms with a stakeholder engagement process. A multilevel approach was implemented for reciprocal engagement between parents of children with congenital anomalies, researchers, health care professionals, and other stakeholders using private and invisible and public Facebook groups, closed Twitter groups, and YouTube. Ethical approval was obtained from Ulster University.

**Results:**

Nonprofit organizations (N=128) were invited to engage with an initial response rate of 16.4% (21/128). Of the 105 parents contacted, 32 entered the private and invisible Facebook groups to participate in the coproduction research. Public Facebook page followers rose to 215, a total of 22 posts had an engagement of >10%, and 34 posts had a reach of over 100. Webinars included requested information on childhood milestones and behavior. YouTube coverage included 106 ConnectEpeople videos with 28,708 impressions. Project information was obtained from 35 countries. The highest Facebook activity occurred during the early morning hours. Achievement of these results required dedicated time management, social media expertise, creativity, and sharing knowledge to curate valuable content.

**Conclusions:**

Building and maintaining a multilayered online forum for coproduction and information sharing is challenging. Technical considerations include understanding the functionality and versatility of social media metrics. Social media offers valuable, easily accessible, quantitative, and qualitative data that can drive the reciprocal process of forum development. The identification and integration of the needs of the ConnectEpeople e-forum was a key driver in the dissemination of useful, meaningful, and accessible information. The necessary dedicated administration to respond to requests and posts and collate data required significant time and effort. Participant safety, the development of trust, and the maintenance of confidentiality were major ethical considerations. Discussions on social media platforms enabled parents to support each other and their children. Social media platforms are particularly useful in identifying common family needs related to early childhood development. This research approach was challenging but resulted in valuable outputs requiring further application and testing. This may be of particular importance in response to COVID-19 or future pandemics. Incorporating flexible, adaptable social media strategies into research projects is recommended to develop effective platforms for collaborative and impactful research and dissemination.

## Introduction

### Background

This is the second paper from the ConnectEpeople project. The first paper reported on project recruitment and findings from coproduction research [1]. This second paper sets out to share the overall learning from the research, technical and ethical obstacles, challenges, and successes in developing the ConnectEpeople e-forum.

An e-forum is defined as a “virtual space for online discussion, allowing deferred participation” [2]. The ConnectEpeople e-forum was an experimental, bespoke web-based community for coproduction research, discussion, information sharing, and dissemination established within social media platforms. The development and management of the e-forum was complex, and limited publications with practical guidance or evaluation methodologies are available. Elliott et al [3] stated that a “gap exists around best practices in establishing, implementing, and evaluating” social media for research purposes. Therefore, the research team’s findings and experiences are reported here to provide practical advice and recommendations for those planning to use social media for health research activities.

### The ConnectEpeople e-Forum

The initial step was to identify the platform on which to host the e-forum. The ConnectEpeople e-forum was intended as a meeting place for stakeholders in the life world of children with one of four congenital anomalies (CAs): congenital heart defects (CHDs), cleft lip with or without cleft palate (CLP), Down syndrome (DS), or spina bifida (SB) from across 9 European countries. A scoping review conducted in 2017 of the most commonly used social media sites by CA and parent support organizations identified more than 97% of CA organizations used web-based communication, with Facebook (82%) and Twitter (56%) being the most popular [4]. In addition, the ease of use and ubiquity of social media distinguished them as ideal platforms for developing e-forums. Social media offer a range of functions to users, that is, creating a presence and identity, information exchange, and as a communication channel to build relationships or communities based on reputation or characteristics [5]. Trust in web-based communities is a direct function of credibility and impartiality [6], traits essential for successful research outcomes. Trustworthy web-based resources enhance viewers’ feelings of reassurance, control, and coping [6].

### Literature Review

The next step was to review the literature to collate current knowledge and recommendations on designing and developing social media–based research. Connecting communities across geographical or institutional boundaries is a fundamental use of information and communication technology [7]. Community informatics includes several methodological pillars, including contexts, values, cases, processes, and systems [8]. Combined with these pillars, frameworks that systematically incorporate sociability and usability into the design and development process are an important element for building a web-based platform [9].

A rapid systematic review of the literature from 2012 to 2020 was undertaken (Multimedia Appendices 1 and 2) to identify papers that described the establishment of a web-based platform for patient, parent, or public and professional communication. CINAHL, MEDLINE (Ovid), Scopus, and hand searches identified 6 papers [10-15] that described the design and establishment of web-based communication platforms. Owens et al [10], Dyson et al [12], Greenwood et al [14], and Han et al [15] engaged with parents, patients, carers, and other stakeholders to generate research questions for children with special needs, respiratory conditions, and people with diabetes. A total of 4 studies used purpose-built websites [10,12,13,15], and 3 studies used social media [11,12,14]. In addition to their website, Dyson et al [12] used Facebook and Twitter to work with parents but with limited success. In contrast, Russell et al [11] used private and invisible Facebook only and established an active, engaged web-based community. Only 1 team had used multiple platforms for separate functions or to engage with different stakeholders, using Facebook, Twitter, Google Hangout, emails, and face-to-face, with considerable success [14]. However, no author has provided recommendations on the most suitable approach for developing a social media–based communication platform. Therefore, process data from the ConnectEpeople project are presented to provide unique insights for researchers planning to establish a multilayered, social media–based research e-forum.

### Objectives

The objectives of this paper are to (1) explore the research, technical, and ethical challenges involved in developing a bespoke, experimental e-forum; (2) identify quantitative and qualitative data collection and analysis methods for social media–based research; and (3) discuss the practical issues of establishing a user-friendly, multicontextual, communication e-forum.

## Methods

### Overview

ConnectEpeople was developed as a complex, adaptive, web-based communication e-forum. It was the beta test of a social media–based network to connect with stakeholders in the lives of children with CHD, CLP, DS, and SB, through Facebook and Twitter as the key communication platforms. The key function of the e-forum was coproduction research and to become a communication and dissemination platform for research and information. There were three key members of the research team (MS, JEMMc, and DE) involved in the design, setup, and running of the ConnectEpeople social media accounts.

As previously reported [1], in the coproduction research stage, 32 research aware parents (RAPs) were recruited from 9 European countries via their parent support organization (n=18), CA registry leader (RL; n=7), ConnectEpeople project survey (n=5), and the project public Facebook page (n=1) and by word of mouth (n=1). On average, parents had two discussions with the researcher before agreeing to participate. The most popular method of meeting the researcher was Skype (n=13), followed by telephone (n=9), WhatsApp video calling (n=8), Facebook messenger (n=1), and FaceTime (n=1). Participants who preferred to use their phones lived in the United Kingdom. The recruitment process took an average of 51 days (SD 40.44), ranging from 6 to 129 days. Completion of the requisite consent form, different time zones across Europe, and children’s health needs were contributing factors.

RAPs joined 1 of 4 condition-specific private and invisible Facebook groups [1]. Private and invisible Facebook groups are invisible to the public, and membership was by invitation only. Using a modified James Lind Alliance approach [16], RAPs in each of the four groups worked with researchers to develop a list of the 10 most important research questions relating to their child’s CA [1] (Multimedia Appendix 3). All RAPs read and signed a social media policy and were offered training to use Facebook and Twitter.

### Building the ConnectEpeople e-Forum

**Figure 1:**
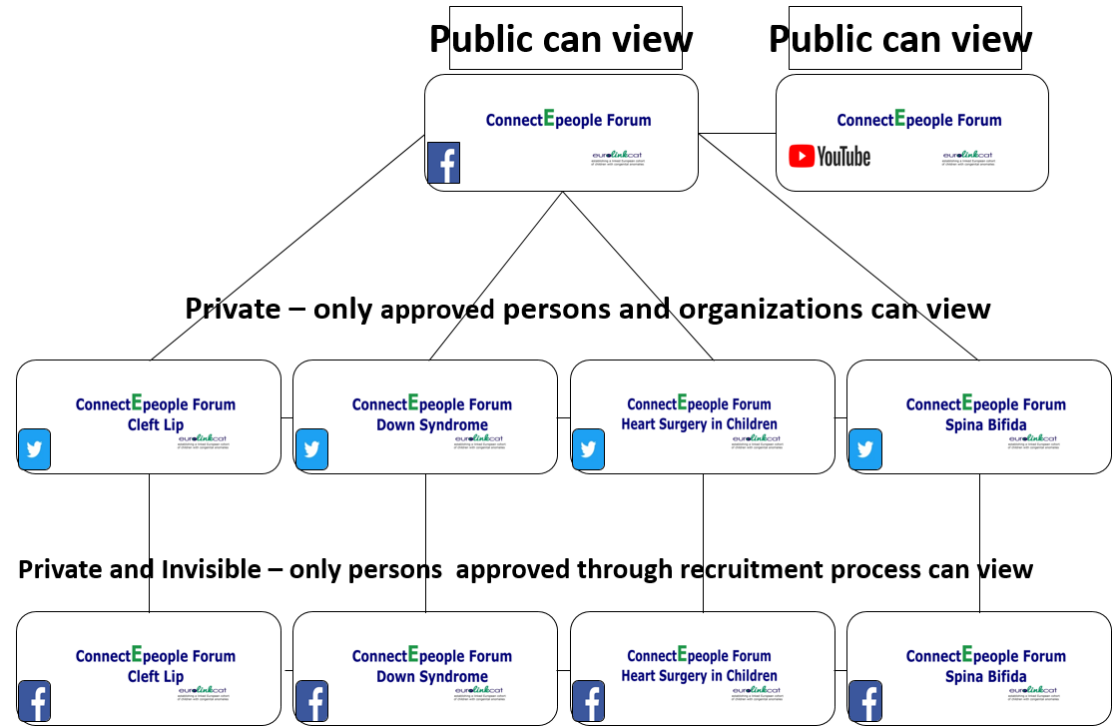
The ConnectEpeople e-forum structure.

The ConnectEpeople social media–based e-forum (Figure 1) was developed to connect stakeholders of children with CHD, CLP, DS, or SB. The e-forum used four CA-specific private and invisible Facebook groups accessible via invitation only to parents of children with CAs engaging in coproduction research. A total of 4 CA-specific closed Twitter groups were accessible to any person requesting to join. A public Facebook page [17] and, as the project progressed, a YouTube channel [18] were accessible to any member of the public.

### Planned Process for Engagement With Stakeholders

The initial plan was to work with RLs across 9 European countries who would act as gatekeepers to connect the research team with local CA organizations, health care professionals (HCPs), and parent support organizations (Multimedia Appendix 4). This process was deemed essential, as they spoke the native language and were attuned to the culture. The intention was for RLs to inform these individuals about the ConnectEpeople project and invite them to engage with the project. An information technology readiness survey carried out with RLs identified the first technical challenge as the results demonstrated that they did not have the necessary social media profile or the internet access required to take part in or facilitate the work of ConnectEpeople. Therefore, parent support organizations across Europe were identified and approached directly via social media by the research team and invited to become gatekeepers for the research study.

### Engaging With Stakeholders

Nonprofit organizations and parent support organizations for CAs across Europe initially identified as part of a scoping review [4] were contacted via email and Facebook messenger and provided with details of the ConnectEpeople project and invited to engage with the research team.

Organizations were invited to engage in four ways:

1. To act as gatekeepers to recruit parents to the ConnectEpeople coproduction research arm
2. To mutually follow Twitter accounts
3. To like, share, and post on the ConnectEpeople public Facebook posts
4. To actively participate in ConnectEpeople webinars

Following the introduction by organizational gatekeepers, potential RAPs were emailed to schedule a screening meeting using Skype, FaceTime, WhatsApp, Facebook Messenger, or telephone. Only those willing to use Facebook could join the project. Parents were able to join the project by contacting the research team through the public Facebook page, following the completion of a project-specific survey, and through contact with RLs.

As a result of the changes in the planned process for stakeholder engagement, the initial recruitment of RAPs was slow. Therefore, the ConnectEpeople survey was developed with RAPs as the first piece of coproduction research. The survey allowed the research team to gather data from a global community of parents of children with CAs and meet the research deadlines for the identification of research priorities.

### Communication With Stakeholders

#### Posting on the Private and Invisible Facebook Groups

Private and invisible Facebook groups were used exclusively to facilitate coproduction research with parents from 7 European countries. Research questions were cocreated, and using an iterative process, the top 10 research priorities were agreed upon [1]. The four private and invisible Facebook groups received the same research questions and information simultaneously. Email was used to communicate information that could not be posted on Facebook, such as large documents. Group posts consisted of research questions, information regarding webinars, updates on the research project, and research activities. RAPs and moderators could freely post in the private and invisible Facebook groups; however, no publicly available hyperlinks were posted to preserve members’ anonymity. Web-based meetings were organized via *Doodle Poll* to meet, discuss, and receive updates on the project, and RAPs could contact the research team directly by email at any time.

#### Posting on Closed Twitter Accounts

For those who wished to follow any of the four closed Twitter accounts, ConnectEpeople sent them a follower request. Membership requests were reviewed by the administrators to ensure legitimacy before acceptance. Twitter accounts demonstrating some activity in their timeline with the corresponding CA were accepted. ConnectEpeople followed all the followers’ accounts. Tweets and retweets were screened to ensure that they were specifically related to research, web-based courses, upcoming events, human interest stories, education, and policy news.

#### Posting on the Public Facebook Page

One public Facebook page was set up to share information and for discussions [17]. Regular posts began on January 7, 2018. Posts were generated by the research team, reposted from organizations followed by ConnectEpeople on Facebook, or identified by the administrators or stakeholders as valid and relevant. No advertisements or calls for donations were reposted, and resources were added to the Facebook public page, including web-based courses and links to research articles.

#### Development of the YouTube Channel and Webinars

Following discussion in the private and invisible Facebook groups and via the project survey, parents identified topics on which they wanted to have more information. This led to the development of the project webinars, giving all stakeholders the opportunity to hear from and engage directly with CA experts from academia, research, and health care. Webinars were held using the videoconferencing software Go To Meeting (LogMeIn), Skype (Microsoft), or Zoom (Zoom Video Communications) and were live streamed. The ConnectEpeople YouTube channel [18] was set up in March 2018 to share project webinars and videos. Webinar videos were cut into short accessible videos and are available to the public on the YouTube channel.

### Data Collection and Analysis

The team collected a wide range of data to determine the most meaningful and impactful information. Qualitative data and feedback from RAPs and other stakeholders and quantitative data, including the number of responses, the time taken to respond, and preferred mode of communication, were recorded. The research team maintained a detailed log of their research, administrative duties and activities, and experiences. The key quantitative outcome measures for the e-forum were metrics data for each of the public social media platforms, as detailed in Textbox 1. The response rates for research-related posts were calculated for the private and invisible Facebook groups.

“Reach is the total number of people who see your content. Impressions are the number of times your content is displayed no matter if it was clicked or not” [19]. Engagement on Facebook is measured by “likes, reactions, comments, shares, and some clicks on links, photos, or videos. Engagement rates on Facebook are measured by engaged users, not total engagements; if someone likes and comments on the post, that counts as two engagements, but one engaged user” [20]. Interactions on Facebook are measured as “communication between an audience member and your...social profile” [21].

**Data collected for each social media platform used in the ConnectEpeople e-forum.**

**Social media platform and the metrics collected**

- Closed Twitter
  Followers
- Public Facebook
  Reach, engagement, views, interactions, and followers
- YouTube
  Views and impressions

### Ethical Considerations

Ethical approval for the study was obtained from the Ulster University, Institute of Nursing and Health Research, Ethics Filter Committee on November 21, 2017.

Only parents who had local social support were recruited to ensure that help was available and accessible should they have become distressed at any point during the project. The project screening process for potential RAPs included completion of the State-Trait Anxiety Inventory (STAI) [22] to limit the risk of any potential emotional burden of taking part in a sensitive research project. Parents provided written informed consent. The use of private and invisible Facebook groups protected the identity and privacy of RAPs and their children.

Posts on the private and invisible and the public Facebook page were reviewed by the administrators before being approved to reduce the risk of inappropriate comments. Any potentially controversial or sensitive comments were discussed among the 3 key research team members for consensus on posting.

## Results

### Engaging Stakeholders

#### CA Organizations

In total, 128 nonprofit and parent support organizations were contacted by email (n=77) and Facebook (n=51). Those contacted by email received 2-3 follow-up messages and 21% (16/77) responded, 1 of whom declined to participate. Of the organizations contacted via Facebook, 10% (5/51) responded, 1 of whom declined the invitation. As the project progressed, email introductions were made by gatekeeper organizations, which facilitated the research team to make new contacts. Response times varied considerably, and 4 of those who responded via Facebook did so within 48 hours and a fifth responded in 59 days. Email responders averaged 72 days (7-365 days).

#### Research Aware Parents

In total, 105 parents were contacted, 54 (51.4%) responded, 38 (36.2%) completed the screening process, and 32 (30.5%) entered the ConnectEpeople private and invisible Facebook groups for CHD (n=4), CLP (n=5), DS (n=13, one RAP dropped out), and SB (n=9). Recruitment was conducted from January 2018 to March 2019 [1].

### ConnectEpeople Private and Invisible Facebook Groups

Over a 19-month period, the research team posted one research-related post per week in the private and invisible Facebook groups. The CHD group was the most active in terms of average number of RAP’s responses to these posts with 54 responses per participant, followed by SB (33.4 responses per participant), CLP (27.2 responses per participant), and DS (7.4 responses per participant). A total of 2 web-based group meetings took place with 13 of 28 and 5 of 28 RAPs responding to *Doodle Polls*, and 4 attended the first meeting and 5 attended the second meeting.

### ConnectEpeople Closed Twitter Group Posts

In total, the 4 closed Twitter groups had 75 followers and followed 650 individuals and organizations.

Two RAPs agreed to follow the closed Twitter groups (SB and CHD). However, the other RAPs did not wish to engage:

I never used Twitter because to me it seems like a spot for weird people with too much time. Sorry but I do not like to test it.

CLP, Germany

No sorry I don’t use any other social media apart from Facebook...spend too much time on here as it is!

CHD, United Kingdom

### ConnectEpeople Public Facebook Data

To date, the ConnectEpeople public Facebook page [17] has 215 followers. One researcher logged on to the public Facebook page daily and posted or reposted information on the four CAs of interest, such as human interest stories, research, public information, and health. All posts were in English, as this was the first language of the researcher. Reposts were from reputable organizations that ConnectEpeople was following. Reposts in languages other than English were first translated using Google Translate. If the researcher could not determine the content following translation, the post was not reposted.

Facebook Insights was used to analyze public Facebook group metrics. Posts with a reach of 100 or above and an engagement rate of 21 or above (10%) were reviewed. Engagement rate was calculated as total engagement or followers × 100 [23]. There were 22 Facebook posts with an engagement of 21 and above, and 34 posts had a reach of 100 and above.

The posts with the greatest reach were those related to project recruitment and survey, which were pinned to the top of the Facebook page. The post with the highest reach (1974) and highest engagement (306) was reposted on *the Mighty* Facebook page and titled “As the school year begins please talk to your kids about disabilities” [24]. *The Mighty* is an online health community created to empower and connect people facing health challenges and disabilities [25]. The ConnectEpeople project–generated Facebook post with the highest engagement (n=132) was one regarding the “ConnectEpeople Research – Parents Voices World Spina Bifida and Hydrocephalus Day 2018” webinar, and the reach was 1282.

**Figure 2:**
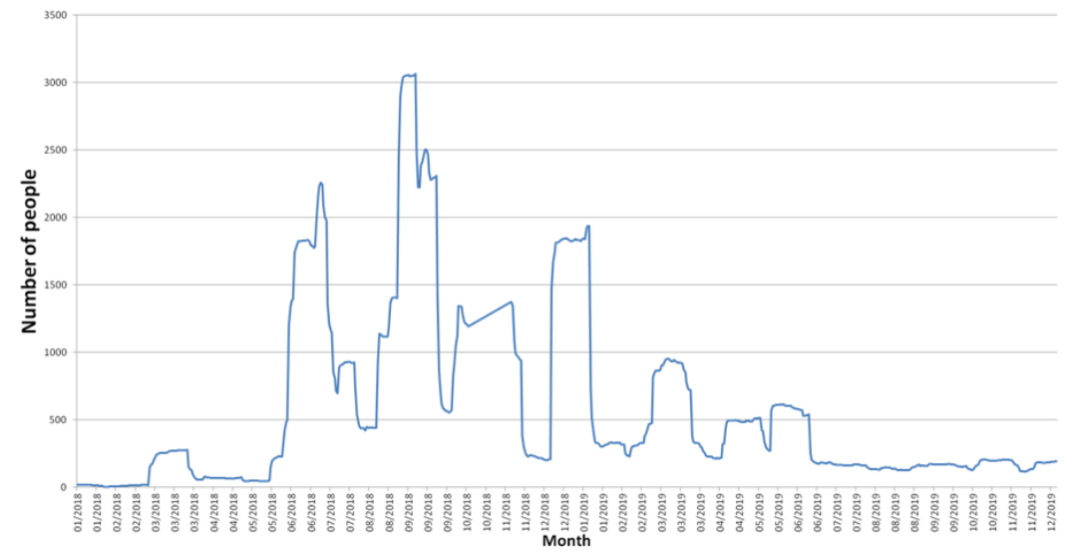
The people for whom any content from the ConnectEpeople public Facebook page entered their screen from January 2018 until December 2019.

Figure 2 shows the number of people who had sight of the public Facebook page. As for all social media projects, the number of people was small (<100) in the early years (January 2018) and increased as the number of interesting posts increased. The recruitment drive in March 2018 shows initial interest, and as posts became more common, additional people viewed the material. The largest number of views (>3000 people) occurred in September 2018. These views were driven by interesting posts or discussions.

**Figure 3:**
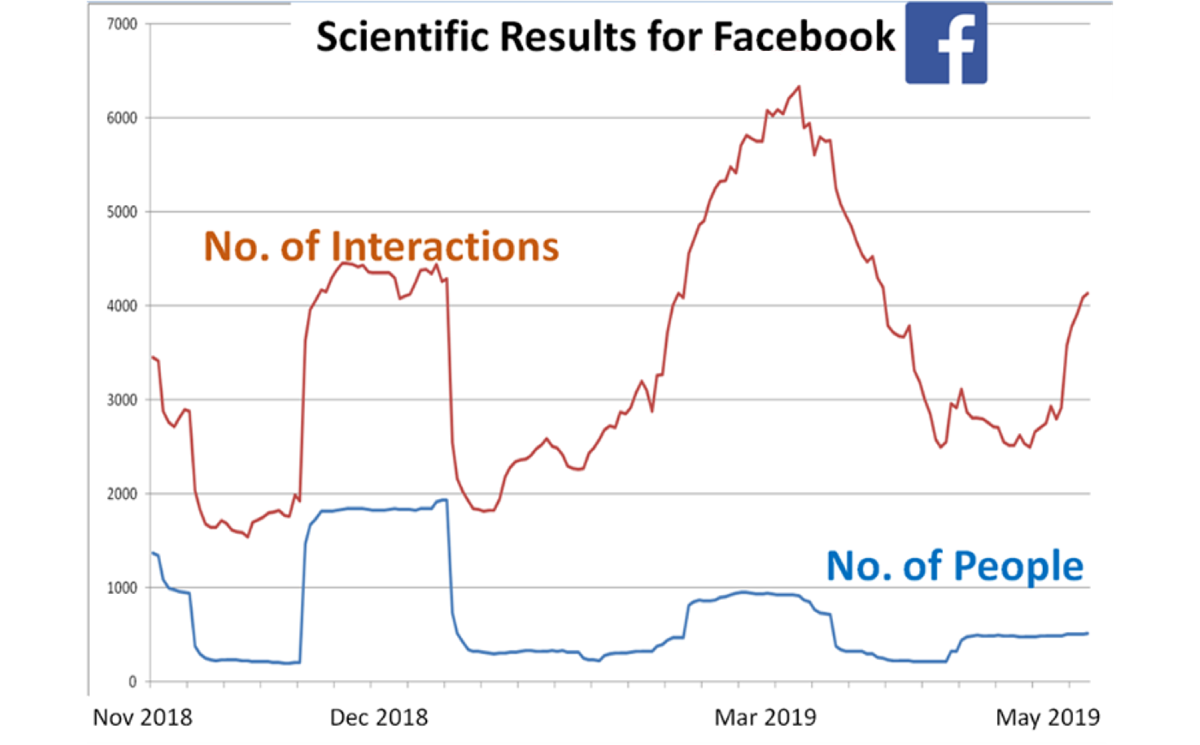
Interactions on the ConnectEpeople public Facebook page.

Figure 3 highlights the number of interactions with different posts, compared with the number of people viewing that post. For example, in December 2018, although almost 2000 people viewed the post, there were more than 4000 interactions, giving an average interaction per person of 2:1. In March 2019, although almost 1000 people viewed the post, there were more than 6000 interactions, giving an average interaction per person of 6:1. Thus, although the number of persons viewing was smaller in March 2019 than in December 2018, the March 2019 post attracted many more interactions (>6000) than the December 2018 post (>4000).

### ConnectEpeople YouTube Channel

The ConnectEpeople YouTube channel currently contains 106 videos. To date, there have been 28,708 impressions for YouTube videos. The most viewed video was one from the World Birth Defects Day 2019 webinar titled “Dr Micaela Notarangelo Breastfeeding for cleft babies WBDD 2019” with 5649 views [26].

### Development of ConnectEpeople Webinars

#### Overview

ConnectEpeople parents wanted to hear more regarding research and surgery, and they asked for more information on their child’s everyday needs. Webinars were developed to provide opportunities to hear from and speak to experts in the CA of interest. These included World Down Syndrome Day 2018 with 2509 people engaging, World Spina Bifida and Hydrocephalus Day 2018 with 6164 people engaging, and World Birth Defects Day 2019 with 1419 people engaging. Webinars with experts in the field of CAs, “Supporting families to enhance their child’s development” by Professor Roy McConkey (educationalist) had 2435 people engaging and “Home monitoring for children with complex heart conditions: new horizons of care for parents, clinicians and researchers” with Professor Frank Casey (consultant pediatric cardiologist) had 2998 people engaging. Those who took part included HCPs, support organization representatives, researchers, and parents. The webinars were cut into short topic-specific videos to promote engagement and posted on the project’s YouTube channel.

#### ConnectEpeople Research Team Members Characteristics

The 3 key members of the research team acted as administrators for the four private and invisible Facebook groups. One team member (DE) set up all on Facebook, Twitter, and YouTube accounts; managed webinars; cut and posted videos to the YouTube channel; and managed the Facebook Insights and metrics collection and analysis. DE also managed the technical aspects of Facebook, Twitter, and YouTube, such as changing banners. One researcher (JEMMc) managed the day-to-day running of the private and public Facebook groups and the 4 Twitter accounts, including screening follower requests on Twitter and posting and responding on Facebook and Twitter. JEMMc also managed contacts and recruitment to the ConnectEpeople project and the development of the webinars. The chief investigator (MS) oversaw the ConnectEpeople social media accounts and made final decisions on all private and invisible Facebook posts and webinar programs. The 3 key researchers were fluent in English only. Team members were available on social media daily from 9 AM to 4 PM and from 7 PM to 10 PM. Facebook and Twitter groups were also checked regularly over weekends and holidays.

#### Additional Findings

**Figure 4:**
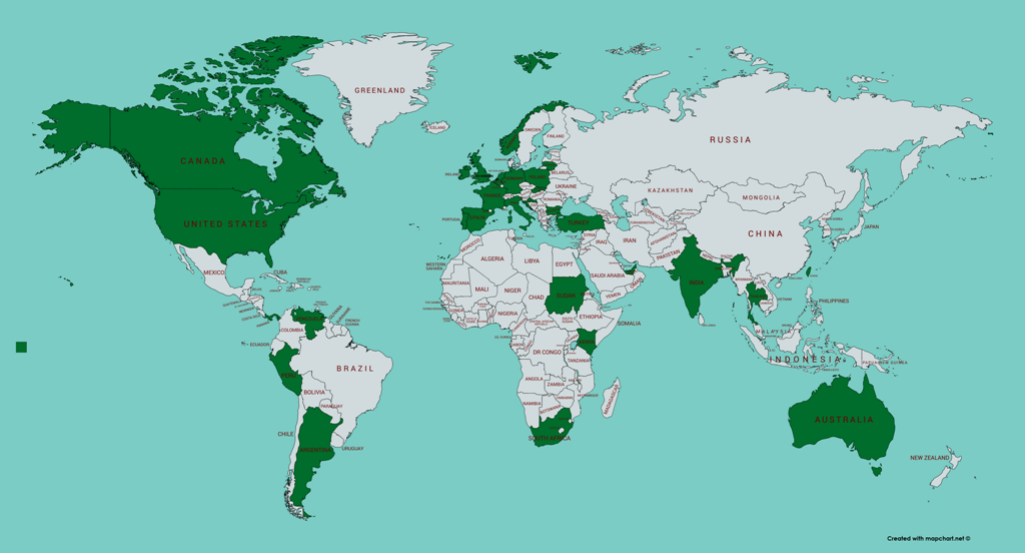
The countries in which ConnectEpeople outputs have been accessed.

Information about ConnectEpeople was accessed by individuals in 35 countries (Figure 4). The most popular time of the day for views on Facebook was in the early hours of the morning with low levels of activity from 2 PM to 11 PM UTC, and on YouTube weekday evenings in line with primetime television. No arguments, negative comments, or inappropriate behaviors were posted on Facebook, Twitter, or YouTube during the project.

## Discussion

### Principal Findings

On the basis of the rapid literature review undertaken and in agreement with Elliott et al [3], there is limited advice for researchers to conduct research based on social media platforms. Building and maintaining the experimental ConnectEpeople e-forum identified a number of interconnected research and technical and ethical learning outcomes for consideration. This may be of particular benefit for teams working with other geographically, culturally, or socially hard to reach groups, such as during the current COVID-19 pandemic. Social media are widely used by stakeholders in children with CAs [4]. Stakeholders were keen to get involved in ConnectEpeople and access new information relating to CHD, CLP, DS, and SB disseminated in a useful, meaningful, and easily accessible way.

Recruitment to the ConnectEpeople coproduction research web-based group was slow because of parents’ family and personal needs. In addition, recruiting RAPs and other stakeholders living across Europe was complicated by the unexpectedly limited bilingual assistance and subsequent *cold calling* on organizations. However, the social media metrics and data collected demonstrate that the e-forum format is an effective and engaging communication platform and safe meeting place.

The ConnectEpeople project investigated the use of social media for research activities, including engagement, recruitment, coproduction research, communication and dissemination, quantitative and qualitative data collection, and creating research impact. Social media have broad applications for research, and the authors recommend incorporating a social media strategy into all research projects. Such a strategy must be developed with the flexibility to adapt and incorporate other platforms as they become available and using feedback from stakeholders. A robust and effective social media strategy requires early financial investment, for while social media are generally free to access and use, considerable time and expertise are necessary to build successful, impactful research communities.

### Research, Technical, and Ethical Considerations

#### Setup of the e-Forum

The ConnectEpeople e-forum was devised as an initial meeting place for geographically distant researchers and stakeholders, and although Elliott et al [3] recommend developing research platforms in collaboration with stakeholders, initial stakeholder input was not possible. Similar to Dyson et al [12], this project was intentionally designed to test multiple social media platforms. Facebook’s greatest function is building relationships [5], and Twitter serves to build a web-based brand or identity. Therefore, these platforms were initially chosen for testing, given their popularity based on the scoping review results. The ubiquity of social media makes them ideal platforms to connect quickly and simply, as many people and organizations have their own accounts and are familiar with making connections via the internet. In addition, Facebook, Twitter, and YouTube are free to join and access. Once contact was made with parents and stakeholders, their views and preferences on communication platforms were sought, leading to the development of the webinars and the YouTube channel.

Lovari et al [27] recommend investment in *multichannel* strategies for web-based communication to effectively reach target populations. During the ConnectEpeople project, text, images, videos, and links were cross-posted on Twitter, Facebook, and YouTube, and information was tailored to the target population’s needs before dissemination. The project saw limited uptake of Twitter groups by RAPs; however, organizations active on Twitter engaged. RAPs focused on engaging in discussions and sharing of information and a more meaningful web-based experience. As Twitter is more aligned with branding, identity, and limited discussion, this may have been a factor influencing use.

Social media–based studies rely on the digital infrastructure. Crucially, for this project before startup, an information technology readiness survey demonstrated that the aims of the project could not be met with the facilities available, leading to a major review of the project plan. Subsequently, the identified digital infrastructure needs were put in place. Digital infrastructure included data storage, access to apps, such as Facebook, Twitter, and YouTube, and additional apps to present webinars and web-based meetings as the project proceeded, such as Zoom. Digital infrastructure also included devices such as computers and mobile phones to enable the research team to have constant access to Twitter and Facebook, which was more active later in the day. Parents were most likely to connect to the internet via their mobile phones, as reported by Pew Research Center [28]. They were also most likely to connect at home. This was ideal for parents to be able to engage when they had free time but difficult to sustain dialog with the research team within working hours. The constant awareness of the project participants, any potential queries or concerns, or the opportunity to engage in sustained meaningful dialog may have led to an increased burden of responsibility for the researcher. It is important that project mobile phones are separate from the researcher’s personal phones and consensus on availability on the web is agreed upon.

#### Recruitment and Engagement With Stakeholders

In this project, RAPs were key partners in identifying research priorities. The engagement and recruitment of parents was expected to take time, as it was difficult to reach groups with limited time availability due to caring for children with complex needs [29]. The initial task of engaging with organizations to act as gatekeepers was also unexpectedly more time consuming. There were a number of reasons for slow uptake identified during conversations with researchers. Organizations were keen to take part; however, many were led by volunteer parents, and time constraints were a major issue. Some organizations required leadership approval to participate; however, many only met biannually, leading to time delays. The key finding was that parents and other stakeholders were rightfully cautious of connecting to the web with groups reporting to be interested in their children. Ensuring participant safety in research poses additional demands when using social media, and Dol et al [30] stated that health researchers require information on “how to ethically use and engage with social media.” Concerns regarding the safety, dignity, and privacy of RAPs and their children led the way for a protracted recruitment process that involved the use of the STAI to check anxiety levels and ensure no additional burden of research on parents. The ConnectEpeople team acknowledged that stakeholders should take the time they needed to ensure they were acting in their child’s best interests. Overall, lack of time was the most common reason given for slow and limited responses in this research, and this reflects that parents who have children with complex health needs have additional concerns and demands on their time.

Organizations also experienced difficulty in finding suitable parents. In addition, only 16.4% (21/128) of the organizations responded. However, in agreement with Russell et al [11] and Han et al [15], the recruitment of parents was most successful when facilitated by trusted third parties, namely, parent support organizations and RLs, as they promoted authenticity. The initial positive personal interaction between the researcher and parents built rapport and trust and encouraged engagement with the project. Using private and invisible Facebook for coproduction was welcomed by RAPs.

#### Communication and Dissemination

The researcher conducting recruitment only spoke English fluently and lived in the United Kingdom and, therefore, relied completely on *cold calling* and strong interpersonal skills to build lasting connections with gatekeepers to facilitate successful recruitment. This also resulted in the necessity of recruiting RAPs who could speak English. The language barrier of pan-European projects and subtleties in language can play a huge role in connecting and communicating successfully on the web. For example, although the translation is available on Facebook, it is only useful for light social discussions and not for those involving technical words and terminology. In addition, cultural aspects and meanings of language can influence the perspectives and understanding of participants.

Good sociability in web-based communities includes the reciprocity and trustworthiness of interactions [31], an important factor in this project. In the ConnectEpeople project, RAPs and stakeholders involved in private and invisible Facebook group discussions were asked to agree to a project-specific social media policy. This was to ensure fair and courteous conduct by members, preserve privacy and confidentiality, and build trust. Clearly defined *rules of engagement* to safeguard individuals have been used for other studies using Facebook [11].

Separate private and invisible Facebook groups were developed for each CA of interest, as research participants trust others with the same life experiences as themselves [32]. However, it was also interesting to find that there were more similarities than differences between the groups. All RAPs wanted up-to-date information; opportunities to talk to experts; and access to appropriate education, health, and social support to enable their children to achieve their maximum potential.

Although clinical concerns play a part of the whole life challenge for children with CAs, they are part of a much wider tableau. Researchers involved in ConnectEpeople were able to connect and discuss with parents directly, which allowed them to learn about the daily life and issues of families who are experts by experience in children with complex health needs. Although the researchers had limited personal experience of CA, they could offer support and information. In a similar way to the web-based community developed by Owens et al [10], “relying on their own humanity and implicit knowledge of what it means to care.” The interaction by the research team in the private and invisible Facebook groups enhanced their knowledge and confidence in selecting and developing suitable posts for the ConnectEpeople public Facebook and Twitter. Importantly, during this project, there were no arguments or negative or inappropriate behaviors on any social media account.

Not all RAPs actively communicated within the groups, and there were clear responders and *lurkers* [33]. Many RAPs were absent from private and invisible Facebook groups for extended periods. During their child’s sickness was understandably a time when many parents were not available. However, for some, the solidarity within the group offered comfort when children were sick in the hospital and far from friends and family, leading to increased activity in their group. Peer-to-peer support is a key feature of online health communities, even when it is not the intended function of the group [10], and Greenwood et al [14] found that seeing others on the web increased engagement. Shared experiences have been identified [34] by users of diabetes web-based forums as valuable tailored advice that they could not acquire from their HCP.

Social media sites provide a platform for sharing information to a wide and varied audience, and messages should be tailored for target audiences [3]. For example, complex information on CAs can be posted and used by those who have experience and insight, such as parents who have a child with a CA or HCP. Developing and instilling trust early on allows users to discuss difficult issues in a safe environment and be confident in the information shared [35]. In this study, many parents reported that they could not access the appropriate help their child needed from a range of providers, including educational and HCPs. Parents also disclosed their feelings of distrust for some health care providers and shared their concerns about being given misleading, inadequate, or inaccurate information and advice. Brady et al [32] identified that internet forum users were concerned about the accuracy of information available on the web and, to a greater extent, the possibility that other users may believe inaccurate information. Identifying and exposing health misinformation being shared on the web has become a major global concern during the COVID-19 pandemic [36]. The ConnectEpeople RAPs actively worked in partnership to produce accurate, engaging, and impactful outputs. RAPs and other stakeholders were reading and downloading information from the ConnectEpeople e-forum. In addition, they created content, for example, webinar videos.

#### Data Collection

ConnectEpeople aimed to identify suitable data collection methods for future research on e-forums based on social media. Qualitative data were available in a number of ways, including contemporaneous notes taken by the researcher during conversations with stakeholders, Facebook and Twitter posts, and consent for recordings of web-based meetings with RAPs, which were transcribed and deleted. All data were stored on password-protected computers.

Social media metrics form the basis of quantitative data and are a source of valuable learning in data management. Metrics data must be collated and stored for analysis, as legacy data cannot be maintained within the Facebook Insights function. It is also important for researchers to understand the functionality of social media metrics and how they can be evaluated and analyzed in relation to research outcome measures and data collection. Analysis of metrics provided insight into project reach and impact. Followers alone, although important for increasing brand awareness, will not enhance the reach of posts. Enhancing engagement should be the key goal of Facebook pages to ensure that messages reach the target audience [37,38]. The findings from the public Facebook page (Figure 2) clearly demonstrate that successful posts are not determined by followers or number of people. It remains incumbent on researchers to identify and share posts that are useful and relevant in a format preferred by the target audience. Klassen et al [39] recommend developing posts that elicit positive feelings and are less serious in tone to increase engagement with followers on Facebook. In their study investigating the content and interaction on a Facebook group related to multiple sclerosis, Della-Rosa and Sen [40] identified that the most popular posts were those on support, information, and awareness. Public Facebook posts generated the highest level of reach and engagement related to promoting positive social interactions for children with a disability attending school [24]. This reflects the outcomes of the ConnectEpeople survey findings and those of the previous ConnectEpeople paper, where parents were very concerned about the psychosocial challenges facing their children [1].

The use of private and invisible Facebook and a public Facebook page provided the level of connectedness required for the different needs of stakeholders. However, there was a limited number of organizations and individuals who could see the project’s Twitter posts, which is likely the reason for the low uptake on Twitter. The research team would recommend single, open Twitter profiles for research projects, which would also reduce the need for cross-posting on Twitter.

#### e-Forum Management

The development of a web-based network is expensive, as it requires ongoing administrative support [41]. Coordinating, reviewing, translating, and responding to posts and connecting to the internet requires considerable investment in time and expertise. Social media accounts are typically uncomplicated to set up; however, updating banners and creating and curating accessible, easy-to-understand, usable, and helpful content to meet the needs of the target audience is challenging. This project benefited from the tremendous support of RAPs, gatekeepers, support organizations, and other stakeholders in the development of content, sharing of ConnectEpeople project details, and actively taking part in webinars. Parents want to promote greater understanding and tolerance of children with complex health conditions to ensure a more positive future for all children.

The overall management of the e-forum required skilled time management, digital infrastructure, and creative skills. Experience and knowledge of different social media platforms were essential to maintain safety on the web, set up and invite RAPs to join the private and invisible Facebook, develop and host webinars for a global audience, and use metrics to demonstrate impact. The key skill required was a thorough up-to-date knowledge of CHD, CLP, DS, and SB. The research team was able to access knowledge in the form of research, testimonials, etc. However, parents and families were the most valuable sources of knowledge regarding the challenges of living with a child with complex health needs. Clinical research was important but so too were social and parenting issues.

Developing social media research that respects and values the knowledge of all, and the reciprocal sharing of perspectives and experiences requires skilled researchers and social media experts to build and maintain internet-based relationships. Although the ConnectEpeople project was aimed at a relatively niche audience, outputs traveled to 35 countries across the world in 2 years. This type of research benefits from global access to social media and the valuable opportunity to facilitate research impact. This may be cultural and attitudinal beliefs, social and societal benefits, enhancing capacity, raising understanding and awareness, and promoting health and well-being [42]. Reach and impact are key components of research, and the power of social media to facilitate this should be included in the planning phase.

#### Other Considerations

The initial project plan to connect with organizations and parents in their country via RLs would still be strongly recommended by the authors to future researchers wishing to replicate our approach. A 2015 Greek study [43] suggested that HCPs and organizations were lagging behind *customers* in their use of social media for health communication, and many researchers are uncertain about using social media for professional activities [44,45]. However, due to the COVID-19 pandemic, support for families has become even more important with the need for strict social distancing, particularly for sick children. This has prompted support for the rapid uptake of social media by support organizations, researchers, and medics [46]. Furthermore, Kemp [47] reported that due to COVID-19, more than 40% of internet users spend more time on social media to help them manage everyday life, and most parents increased their use of social media for information and social support [48]. Many international organizations now use social media to publicize their work and disseminate information, for example, the World Health Organization, United Nations Children’s Fund, Centers for Disease Control and Prevention, European Commission, and the International Clearinghouse for Birth Defects. Social media is evolving as a credible and sustainable choice for engagement and research.

### Future Considerations for the e-Forum

The model by Young [49] for the life cycle of web-based communities consists of four stages, namely inception, establishment, maturity, and mitosis. This paper has discussed the ConnectEpeople e-forum up to the establishment stage, where the activities primarily concerned making connections and building a core group of active members. Social media–based researchers must consider how to adapt as groups grow and progress through maturity and mitosis and how changes or increase in user shared content, disengagement, or potential splinter groups should be managed and the likely impact of this on their research.

As research e-forums are developed, understanding the life cycle of such web-based communities is important to guide and direct research endeavors and facilitate continued engagement. Meeting the future needs of members may include the use of different web-based activities, such as blogs and podcasts, to promote the transfer of knowledge and practice and encourage a diversity of membership. Furthermore, other research teams have reported parents and experts by experience can successfully take ownership and become leaders and drivers of the e-forum they have helped to build [10,11].

The COVID-19 pandemic has resulted in new global health needs, including those of children with CAs and their families. Researchers can efficiently and effectively learn from active research e-forums to codevelop research, engage in timely patient and public involvement in research, and be leaders in time-sensitive research. This ensures that the e-forum continues to meet the evolving needs of members and is relevant long term. In addition, the social media use of the target audience should continually be reviewed as new social media platforms become popular.

### Limitations

There were only 2 administrators managing public Facebook, four private and invisible Facebook groups, and 4 closed Twitter groups content. The administrators’ first language was English, limiting the availability of multilingual posts on social media and connecting with individuals across Europe. A number of videos posted on the public Facebook page did not have available organic video metrics due to an issue experienced by Facebook from October 25 to 28, 2019, which may have had an impact on the calculated reach and engagement with some posts. Challenges exist with drawing conclusions surrounding the potential impact on families and children’s health, as it is difficult to track the use and implementation of messages shared on social media. In addition, the impact of technology poverty or limited access to digital infrastructure on recruitment and engagement has not been investigated.

### Conclusions

Effective use of social media by researchers and relevant key stakeholders requires an understanding of their unique functions and careful planning in design, management, and evaluation strategies. Social media as a research tool has enormous potential to connect and empower people and reach new audiences while providing valuable data. COVID-19 has been a catalyst in the rapid and likely enduring uptake of social media for health information provision by members of the scientific and medical communities [46]. When social distancing measures due to COVID-19 are reduced, hybrid models of research are likely to become commonplace, combining web-based and in-person social connections. Therefore, developing web-based research skills and techniques to harness the versatility of social media has become an essential tool for researchers. The development of a framework for social media research recommended by Elliott et al [3] would require flexibility and ongoing re-evaluation to facilitate the life cycles of social media groups.

## Appendix

Multimedia Appendix 1 Systematic literature search.

Multimedia Appendix 2 PRISMA (Preferred Reporting Items for Systematic Review and Meta-Analyses) diagram for systematic literature search for research articles investigating the design and establishment of a web-based communication platform for patient, parent, or public and professional communication.

Multimedia Appendix 3 Ten most important research questions of ConnectEpeople participants with children who have Down syndrome, spina bifida, cleft lip with or without cleft palate, and congenital heart defects.

Multimedia Appendix 4 Original engagement plan for the ConnectEpeople project.

## Abbreviations

CA: congenital anomaly
CHD: congenital heart defect
CLP: cleft lip with or without cleft palate
DS: Down syndrome
HCP: health care professional
RAP: research aware parent
RL: registry leader
SB: spina bifida
