# Supplementary material for: Using Social Media as a Research Tool for a Bespoke Web-Based Platform for Stakeholders of Children With Congenital Anomalies: Development Study
Source: JMIR Pediatr Parent. 2021 Nov 15;4(4):e18483. doi: 10.2196/18483 (PMC8663440; doi:10.2196/18483)
Supplement: Multimedia Appendix 1 [file pediatrics_v4i4e18483_app1.docx]

**Appendix 1**

**Systematic Literature Search**

Search terms

set*-up

establish*

design*

develop*

buil*

creat*

eforum*

e-forum*

“electronic forum*”

chat room*

discussion room*

discussion board*

message board*

“virtual communit*”

“online communit*”

“digital communit*”

Health*

Patient*

Parent*

e-patient*

Years 2012-current

English language
