# Supplementary figures and images for: Using Social Media as a Research Tool for a Bespoke Web-Based Platform for Stakeholders of Children With Congenital Anomalies: Development Study
Source: JMIR Pediatr Parent. 2021 Nov 15;4(4):e18483. doi: 10.2196/18483 (PMC8663440; doi:10.2196/18483)

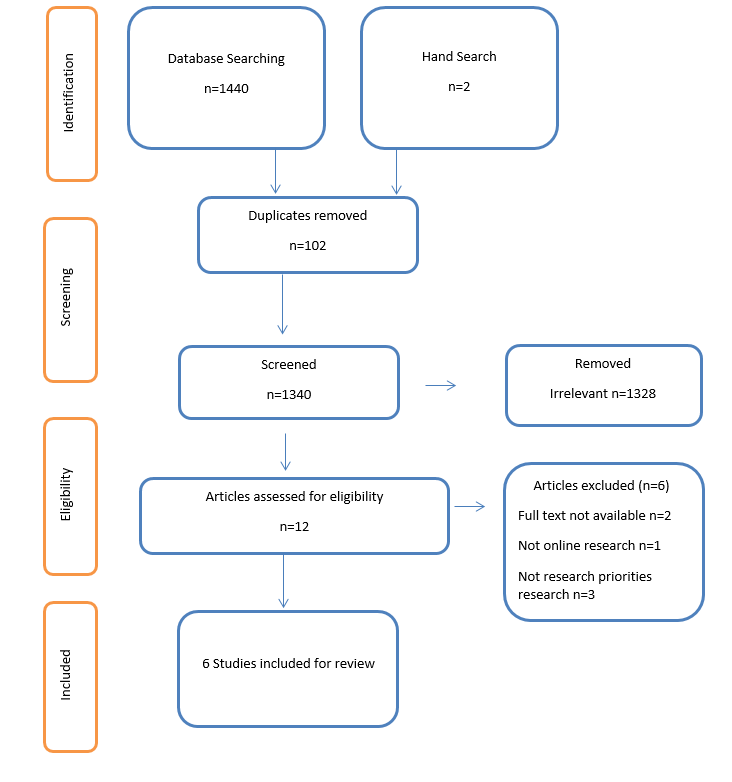

Supplement: Multimedia Appendix 2 [file pediatrics_v4i4e18483_app2.png]

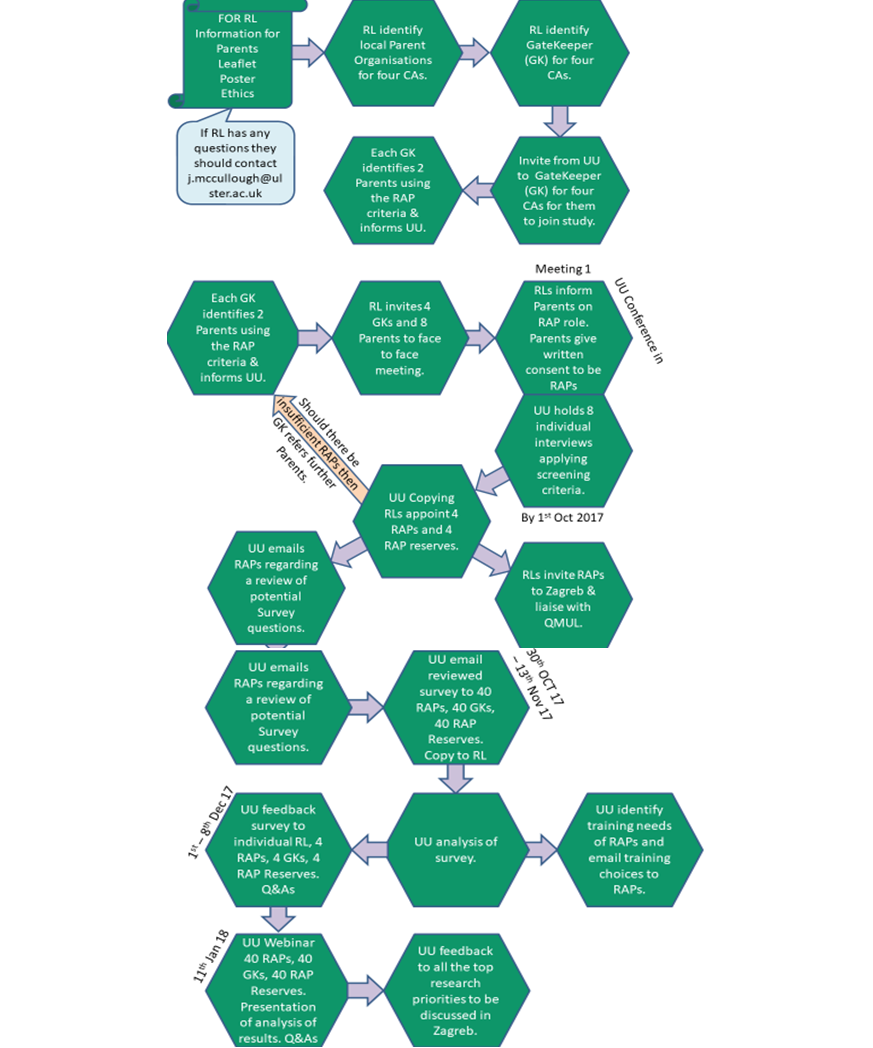

Supplement: Multimedia Appendix 4 [file pediatrics_v4i4e18483_app4.png]
