# Supplementary material for: Using Social Media as a Research Tool for a Bespoke Web-Based Platform for Stakeholders of Children With Congenital Anomalies: Development Study
Source: JMIR Pediatr Parent. 2021 Nov 15;4(4):e18483. doi: 10.2196/18483 (PMC8663440; doi:10.2196/18483)
Supplement: Multimedia Appendix 3 [file pediatrics_v4i4e18483_app3.docx]

## **Appendix 3**

## **Table a. Ten most important research questions of ConnectEpeople participants with children who have Down Syndrome (n=35)**

| **Question** | **Really important/ Important n (%)** | **Not sure n (%)** | **Definitely not important n (%)** |
| --- | --- | --- | --- |
| How can I maximize my child's educational attainment? | 34 (97) | 1 (3) | 0 (0) |
| What dietary supplements should my child be taking? | 34 (97) | 1 (3) | 0 (0) |
| Does exercise enhance the immune system of children with Down Syndrome? | 33 (94) | 1 (3) | 1 (3) |
| Would early intervention e.g. tummy time, creeping and crawling, enhance my child’s development? | 33 (94) | 1 (3) | 1 (3) |
| How many children, with the same condition as my child, go to mainstream school? | 32 (91) | 1 (3) | 2 (6) |
| Where would I find specialised information such as video clips of parents feeding a baby with my child’s condition? | 32 (91) | 0 (0) | 3 (9) |
| Is obesity a problem with my child's condition? | 32 (91) | 0 (0) | 3 (9) |
| What is the latest genetic research relating to my child's condition? | 32 (91) | 3 (9) | 0 (0) |
| What is the psycho-social impact of my child's condition on my child, and our family? | 32 (91) | 1 (3) | 2 (6) |
| What complementary therapies are beneficial for my child? | 32 (91) | 2 (6) | 1 (3) |

## **Table b. Ten most important research questions of ConnectEpeople participants with children who have Spina Bifida (n=39)**

| **Question** | **Really important/ Important n (%)** | **Not sure n (%)** | **Definitely not important n (%)** |
| --- | --- | --- | --- |
| How many children have surgery and how many survive? | 37 (95) | 2 (5) | 0 (0) |
| What is the psycho-social impact of my child's condition on my child, and our family? | 37 (95) | 2 (5) | 0 (0) |
| What is the normal milestone development for a child with the same condition as my child? | 36 (92) | 2 (5) | 1 (3) |
| If my child has to take time out of school will their education continue? | 36 (92) | 3 (8) | 0 (0) |
| How can I maximize my child's educational attainment? | 36 (92) | 3 (8) | 0 (0) |
| What complementary therapies are beneficial for my child? | 36 (92) | 2 (5) | 1 (3) |
| What dietary supplements should my child be taking? | 35 (90) | 3 (8) | 1 (2) |
| What devices or products are the best to buy for my child at different life stages? | 35 (90) | 3 (8) | 1 (2) |
| How many children, with the same condition as my child, go to mainstream school? | 35 (90) | 3 (8) | 1 (2) |
| What type of operations are available for babies in the womb to reduce the effect of their condition? | 34 (87) | 4 (10) | 1 (3) |

## **Table c. Ten most important research questions of ConnectEpeople participants with children who have Cleft Lip with or without cleft Palate (n=16)**

| **Question** | **Really important/ Important n (%)** | **Not sure n (%)** | **Definitely not important n (%)** |
| --- | --- | --- | --- |
| What is the rate of re-occurrence of cleft lip with or without cleft palate among siblings? | 16 (100) | 0 (0) | 0 (0) |
| Are there lactation consultants with expertise in supporting parents who have a child like mine? | 16 (100) | 0 (0) | 0 (0) |
| What are the genetic and environmental causes of cleft lip with or without cleft palate? | 16 (100) | 0 (0) | 0 (0) |
| Where would I find specialised information such as video clips of parents feeding a baby with my child’s condition? | 16 (100) | 0 (0) | 0 (0) |
| What is the best age for children with a cleft to have surgery? | 16 (100) | 0 (0) | 0 (0) |
| What is the latest genetic research relating to my child's condition? | 15 (94) | 1 (6) | 0 (0) |
| How can I maximize my child's educational attainment? | 14 (88) | 2 (12) | 0 (0) |
| What complementary therapies are beneficial for my child? | 14 (88) | 2 (12) | 0 (0) |
| What is the psycho-social impact of my child's condition on my child, and our family? | 14 (88) | 0 (0) | 2 (12) |
| What is the normal milestone development for a child with the same condition as my child? | 13 (81) | 2 (13) | 1 (6) |

## **Table d. Ten most important research questions of ConnectEpeople participants with children who have Congenital Heart Defects (n=10).**

| **Question** | **Really important/ Important n (%)** | **Not sure n (%)** | **Definitely not important n (%)** |
| --- | --- | --- | --- |
| If my child is diagnosed with a heart condition in the womb are there any medications I can take to help my baby? | 10 (100) | 0 (0) | 0 (0) |
| Is it ok for my child to get vaccinated? | 10 (100) | 0 (0) | 0 (0) |
| Is there an increased number of hospital admissions during winter with children with heart defects? | 10 (100) | 0 (0) | 0 (0) |
| Is obesity a problem with my child's condition? | 10 (100) | 0 (0) | 0 (0) |
| What is the latest genetic research relating to my child's condition? | 10 (100) | 0 (0) | 0 (0) |
| How can I maximize my child's educational attainment? | 10 (100) | 0 (0) | 0 (0) |
| What is the psycho-social impact of my child's condition on my child, and our family? | 10 (100) | 0 (0) | 0 (0) |
| Can you pick up heart defects during pregnancy and reduce the damage? | 9 (90) | 1 (10) | 0 (0) |
| How many children have heart surgery and how many survive? | 9 (90) | 1 (10) | 0 (0) |
| What age is my child likely to live to? | 9 (90) | 1 (10) | 0 (0) |
